# Performance assessment of disposable carbon-based immunosensors for the detection of SARS-CoV-2 infections

**DOI:** 10.1038/s41598-025-92104-7

**Published:** 2025-03-05

**Authors:** Olga L. Agudelo, Vanessa Reyes-Loaiza, Lina Giraldo-Parra, Mariana Rosales-Chilama, Sammy Perdomo, María Adelaida Gómez, John W. Rodriguez, Viviana Ortega, Carlos F. Daza Rivera, Diana Galindo, Drochss P. Valencia, Mauricio Quimbaya, Simón Plata, Robert Bogdanowicz, Fernando Rosso, Andres Jaramillo-Botero

**Affiliations:** 1 Centro de Investigaciones Clínicas (CIC), Fundación Valle del Lili, Cra 98 No. 18 – 49, 760032 Cali, Colombia; 2 iOMICAS, Pontificia Universidad Javeriana, Calle 18 # 118-250, Cali, Colombia; 3 Centro Internacional de Entrenamiento e Investigaciones Médicas (CIDEIM), Cali, Colombia; 4 Universidad ICESI, Cali, Colombia; 5 Gobernación del Valle del Cauca, Secretaria de Salud Departamental, Laboratorio de Salud Pública Departamental del Valle, Cra. 76 #4-70, Cali, Valle del Cauca Colombia; 6 Faculty of Electronics, Telecommunications and Informatics, Gdansk University of Technology, Narutowicza Str.11/12, 80-233 Gdansk, Poland; 7 Chemistry and Chemical Engineering, California Institute of Technology, 1200 E California Blvd, Pasadena, CA 91125 USA

**Keywords:** SARS-CoV-2, Biosensor, Immunoassays, Antigen, Spike protein, Point-of-care testing, Immunochemistry, Viral infection, Graphene, Biosensors, Nanobiotechnology, Infectious diseases, Biotechnology

## Abstract

We designed, developed, and clinically tested two rapid antigen-based immunosensors for SARS-CoV-2 detection, enabling diagnosis and viral load quantification for under USD $2. In a first clinical study, a screen-printed disposable carbon-based (SPC) sensor was assessed on prospectively recruited adult participants classified into three study groups: healthy donors (n = 46); SARS-CoV-2-infected symptomatic patients (n = 58); and co-habitants of patients without prior testing (n = 38). Nasopharyngeal aspirates (NA), oropharyngeal swabs (OS), and saliva (SA) samples were obtained from all participants. Performance was measured in terms of clinical sensitivity and specificity against a reference diagnostic RT-qPCR kit and analytical sensitivity (limit of detection, LoD) and specificity using recombinant material in lab tests. A second study was performed using the same sensor design, albeit with laser-induced graphene (LIG) electrodes, using nasopharyngeal swabs (NS) on 224 patient samples obtained at different stages of the pandemic, of which 110 tested negative and 114 positive via RT-qPCR. We find OS was the most informative sample, when compared to NA and SA. The SPC-based sensors had a 93.8% sensitivity and 61.5% specificity with OS samples, while the LIG-based sensors with NS had a lower sensitivity of 68.93%, albeit a significantly higher specificity of 86.17%. We believe specificity values for the SPC sensors were driven by positive results from co-habitants and healthy donors and were affected by the low sensitivity (75.5%) and high LoD (> 20,000 viral copies/mL) of the reference RT-qPCR kit used, and the lower sensitivity of the LIG-based was due to a reduced set of effective antigen-binding sites caused by the non-covalent LIG-mAb ligands used. The immunosensor’s LoD to spike protein in phosphate-buffered saline (PBS) for both types of sensors was near 1 fg/mL and showed no cross-reactivity to recombinant structural proteins of Epstein-Barr and Influenza. Performance metrics and time-to-result (5 < 12 min) provide proof-of-principle of the immunosensor’s applicability as a low-cost, rapid technology for determining SARS-CoV-2 infections. Changing the working electrode material to LIG, instead of SPC, improved specificity even in the presence of pathogen variants. Discordant results between our two immunosensor versions and RT-qPCR tests are attributed not only to limited antibody effectiveness in the former but also to the quality of RT-qPCR probes used at the height of the pandemic.

**Supplementary Information:**

The online version contains supplementary material available at 10.1038/s41598-025-92104-7.

## Introduction

The SARS-CoV-2 pandemic marked one of the most severe global health crises in modern history^1^, resulting in millions of deaths and significant disruptions to our quality of life due to its widespread economic and social impacts^2–4^. Since 2019, the scientific community has made tremendous efforts to combat the virus and its variants^5–7^, most notably through the development of vaccines that have been instrumental in suppress viral transmission^8,9^. In parallel, innovative diagnostic techniques have emerged to provide early, cost-effective alternatives for virus detection^10–13^. These advancements aim to enable accurate, rapid testing and prevent future waves of infection^14^. However, the current clinical ‘gold standard’ for molecular viral diagnostics, reverse-transcriptase quantitative PCR (RT-qPCR), remains limited by high costs and lengthy processing times (3–4 h for results), making it impractical for large-scale use in many settings^15^.

Although RT-qPCR technologies can offer high sensitivity and specificity, they are confounded by poor sampling and sample manipulation, choice of primers, probes, and reagents, the emergence of gene target variants, and the prevalence of co-infections, which can interfere with test specificity. Furthermore, RT-qPCR requires isolation and amplification of the viral RNA, as well as the use of expensive instrumentation resulting in high test costs ($100–200), which restrict its ubiquitous application, hence hindering effective control strategies that could break the chain of infections, especially in resource-constrained settings^16^.

Today, a wide range of alternative technologies are available to diagnose viral diseases, including immunoassays^17^ and biosensors based on immunoassays^18–21^. Traditional immunoassays measure the presence of an analyte in a solution through the use of antibodies or antigens, by producing a measurable signal in response to the binding of an antibody to its antigen. Novel biosensors that translate the presence of viral material into measurable changes in a material or device’s physicochemical properties have recently been developed for SARS-CoV-2 diagnosis. Transduction of viral loads using field-effect currents^22^, surface plasmon energies^23,24^, optical shifts^23^, electrochemical processes^25–27^, piezoelectric^28^ or thermal fluctuations^23^ have been successfully demonstrated. Yet, most of these remain at a low technology readiness level (TRL < 4) or are limited by poor scalability.

This work introduces a novel antigen-based diagnostic platform, based on disposable carbon-based 3-electrode sensors, with an analytical limit of detection of 1 fg/mL for SARS-CoV-2 spike glycoproteins^29^, offering rapid screening capabilities with results available in just 5–12 min. Furthermore, two distinct sensor designs were clinically validated using real-time and batch samples obtained during the COVID pandemic: a nanostructured screen-printed carbon (SPC-based) sensor and an innovative laser-induced graphene (LIG-based) sensor. Both designs are disposable, scalable, and cost-effective, with production costs below $2 (as shown in Table S2 of the SI), establishing a significant advancement in affordable and rapid diagnostics for resource-limited settings. Both types of sensors were functionally validated using cyclic voltammetry (Fig. S2 of the SI) and electrochemical impedance spectroscopy (Figs. S1 and S3 of the SI). Both types of sensors provide a linear charge transfer resistance response (Fig. S4A and C of the SI), enabling precise tracking of viral load concentrations and their temporal dynamics^30^—a feature not commonly reported in existing diagnostic tools (including lateral flow devices). Rigorous *in-vitro* validation was conducted using positive and negative controls, leveraging recombinant structural proteins specific to SARS-CoV-2, influenza, and Epstein-Barr virus (EBV), confirming exceptional selectivity (Fig. S4B and D of the SI).

## Materials and methods

### Ethics statement

This study was approved and monitored by the institutional review board for the ethical conduct of research involving human subjects of Fundación Valle del Lili (FVL), under code #1594, in accordance with both national (resolution 008430, República de Colombia, Ministry of Health, 1993) and international (Declaration of Helsinki and amendments, World Medical Association, Fortaleza, Brazil, October 2013) guidelines. All participants voluntarily took part in the study, and written informed consent was obtained from each participant.

### Study participants

As mentioned, two separate clinical studies were conducted to validate the sensor technology for SARS-CoV-2 detection. The first study, performed at FVL, evaluated the SPC-based disposable sensors. The second study, conducted at the Laboratorio de Salud Pública Departamental del Valle del Cauca (LSPDV), assessed the LIG-based disposable sensors. At FVL, the study included adult participants (≥ 18 years), both men and women, recruited based on the defined inclusion criteria for any of three study groups: a) healthy individuals (n = 46), all of whom had no history or symptoms of COVID-19 and tested negative for SARS-CoV-2 via RT-qPCR; b) SARS-CoV-2-infected and symptomatic patients (n = 58), who were confirmed to have SARS-CoV-2 infection through RT-qPCR testing of nasopharyngeal aspirate samples and presented clinical signs and symptoms of COVID-19 according to the Colombian national diagnostic and treatment guidelines, and c) a subset of asymptomatic co-habitants (n = 38) of the symptomatic patients recruited within group b . The LSPDV study analyzed a total of 224 samples, obtained from individuals at various stages of the pandemic. These included 110 negative samples and 114 positive samples, confirmed via RT-qPCR.

For all participants, demographic and clinical data were obtained directly from clinical records and stored in a RedCAP database (Supplementary Material, Table S2). 3 samples were collected from each participant in the first clinical study at Fundación Valle del Lili in Cali, Colombia, one nasopharyngeal aspirate, one oropharyngeal swab, and one saliva sample. For the second clinical study, all samples were obtained via oropharyngeal swabs, and collected at the LSPDV.

### Specimen collection and storage

Nasopharyngeal aspirates were collected following standard techniques and World Health Organization (WHO) guidelines^31^. For the protocol followed at FVL, approximately 10 mL of the sample was placed in saline solution (0.9% NaCl) and stored in appropriate biological sample containers for transport to the microbiology laboratory, where RT-qPCR for SARS-CoV-2 detection was performed. 300 µL of the original sample was transferred into an Eppendorf tube containing 300 µL of PBS with 0.5% Triton X-100. Oropharyngeal swab samples were obtained by gently rubbing flexible sterile polyester or Dacron® swabs on the oropharyngeal mucosa^31^. The swabs were immediately placed in an Eppendorf tube containing 400 µL of PBS with 0.25% Triton X-100. The swab stem was cut off, and the tube was capped. For saliva collection, participants were instructed to refrain from eating, drinking, smoking, or using oral hygiene products for at least 10 min before sampling. Approximately 500 µL of saliva from the oral cavity was aspirated using a sterile plastic Pasteur pipette and dispensed into an Eppendorf tube containing 500 µL of PBS containing 0.5% Triton X-100. All specimens were transported and stored at 4 °C and processed at the biosafety laboratory in CIDEIM within 48 min of collection.

All samples from the LSPDV were stored in viral transport medium (VTM) and kept at -70 °C after collection and maintained at a controlled temperature of 4 °C for processing.

### Bradford assay for protein quantification

We characterized the quality of sampling during each clinical assessment study, in terms of protein content, for three different methods used during the first study, namely nasopharyngeal aspirate, oropharyngeal swab, and saliva, and the oropharyngeal samples used during the second study. This was done upon arrival using a modified Bradford assay^32^. The standard curve was generated using a five-fold serial dilution of 1.25 mg/mL Bovine Serum Albumin (BSA) (Sigma-Aldrich Cat. A9647). Absorbance measurements were taken at 595 nm using a Dinex microplate reader.

### Protein separation by polyacrylamide gel electrophoresis

Solubilized proteins were separated by molecular weight using sodium dodecyl sulfate–polyacrylamide gel electrophoresis (SDS-PAGE) with a BioRad Mini-PROTEAN II® electrophoresis system (USA). Samples were loaded onto 10–12% polyacrylamide gels and subjected to electrophoresis at a constant current of 100 mA for 2 h in SDS running buffer (25 mM Tris base, 0.2 M glycine, 0.1% SDS). To calibrate molecular weights, Precision Plus Protein™ Kaleidoscope™ Prestained Protein Standards (BioRad, cat. 1610375) were included. Protein bands were visualized using silver staining, and digital images of the gels were captured using the Gel Doc EZ documentation system and its associated software (BioRad).

### Reference standard

RT-qPCR served as the reference standard for molecular diagnosis. Nasopharyngeal aspirate samples collected at FVL were analyzed using the ACCUPOWER SARS-COV-2 RT-PCR kit (Bioneer, South Korea), which targets the detection RdRp and E gene transcripts. At the LSPDV, two methodologies were used for virus identification. The first utilized the BD MAX-SARS-CoV-2 SYSTEM (catalog 442827), an automated system that performs viral RNA extraction and amplification via real-time PCR (rRT-PCR) to detect the N1 and N2 genes of SARS-CoV-2. The second methodology, used the Berlin Protocol version 2 (Diagnostic detection of 2019-nCoV by real-time RT-PCR) to detect the Gen E and RdRp-2 genes. Viral RNA was extracted using the Nucleic Acid Extraction Kit (TANbead®). Amplification was performed using the SuperScript III Platinum One Step qRT-PCR enzyme kit, and reactions were carried out on the Applied Biosystems 7500 FAST Dx Real-Time PCR System and the LightCycler® 96 Instrument from Roche.

### Fabrication and Functionalization of the electrodes

**Fig. 1:**
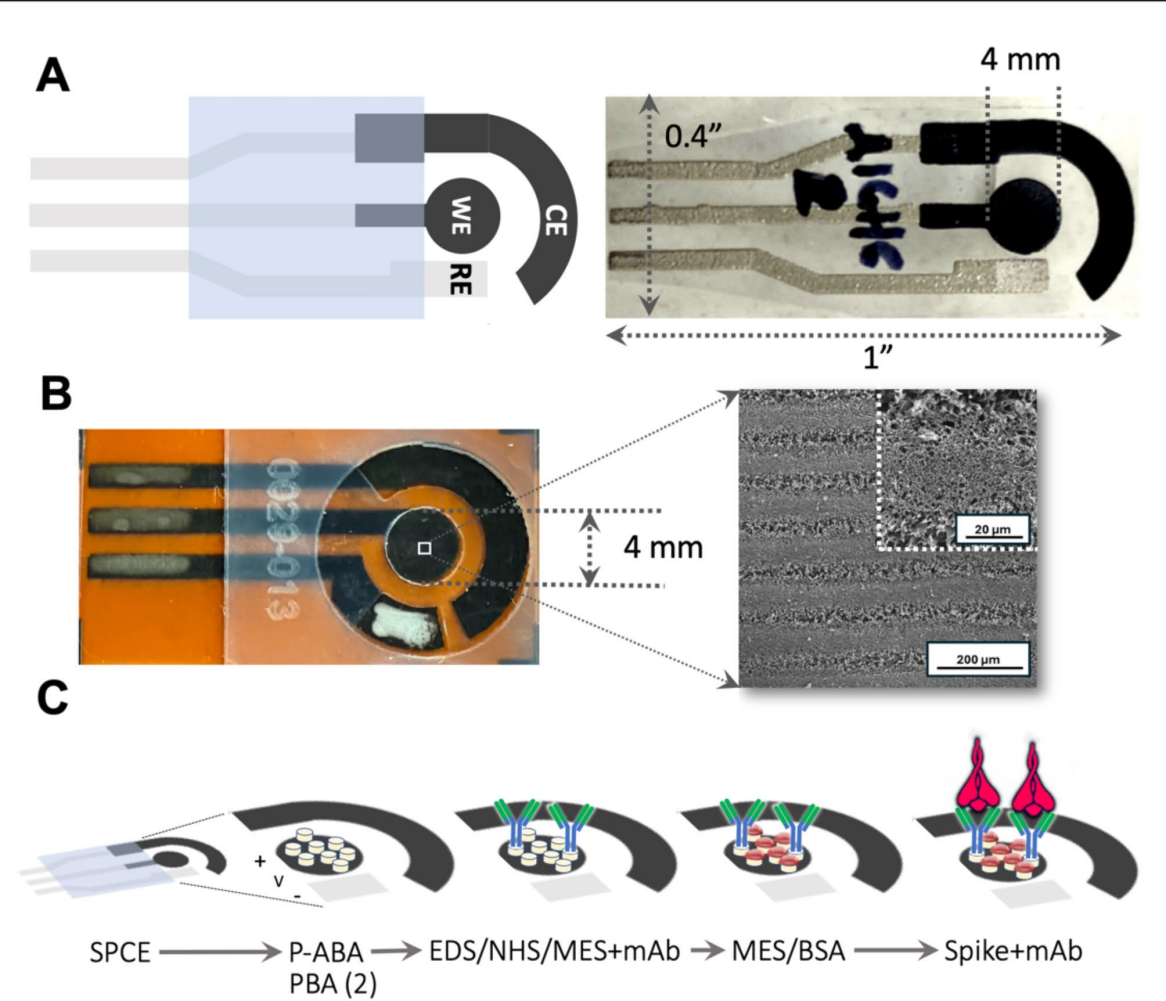
(**A**) Screen-printed carbon-based electrodes (SPCs) used in first clinical assessment study. (Left) The WE and CE are made of carbon paste and the RE is made of Au/AuCl, as well as the grey terminal contacts. The shaded blue area corresponds to an insulating dielectric layer (ESL ElectroScience Europe, UK, ESL 4917). Each SPC-based unit measures ~ 1 × 0.4″ (not shown to scale) with a WE having radius 4 mm. (Right) photograph of an actual SPC-based sensor. SPC-based sensors reported here were fabricated at the Universidad del Valle facilities, in Cali, Colombia. (**B**) (Left) Photograph of laser-induced graphene-based (LIGs) sensor used in the second clinical assessment study. All electrodes are made of LIG, with the RE covered with Au/AuCl, as well as the grey terminal contacts. A transparent polyethylene terephthalate (PET) overlay mask acts as the insulating dielectric layer. Each LIG sensor unit measures ~ 1 × 0.4″ (not shown to scale) with a WE having radius 4 mm. LIG-based sensors reported here were fabricated at the iÓMICAS Research Institute facilities of the Pontificia Universidad Javeriana, in Cali, Colombia. (Right) Scanning electron micrograph image of the LIG surface at 619 × and 4kx magnifications shows highly porous structure. (**C**) SPC/LIG functionalization for SARS-CoV-2 spike protein detection. From left: bare electrode, para-aminobenzoic acid electrodeposition (PABA) using cyclic voltammetry for SPC and pyrene butyric acid (PBA) drop-casting for LIG, covalent attachment of carboxylic group from surface PABA to SARS-CoV-2 spike protein monoclonal antibody (mAb), passivation of open carboxylic sites on PABA surface with BSA, and ligation of spike proteins from patient’s sample to antibodies.

SPC-based electrodes were fabricated using a thick film screen-printing deposition process. Each SPC consisted of a working electrode (WE, with a diameter of 4 mm), a reference electrode (RE), and a counter electrode (CE), arranged in an interdigitated layout (Fig. 1A) on a polyethylene terephthalate (PET) film substrate. The WE and CE were printed using DuPont’s BQ221 carbon-based conductive paste (DuPont, USA) and cured at 130 °C for 10 min. The RE was screen-printed using DuPont’s 5874 Ag/AgCl paste (DuPont, USA), with an Ag:AgCl ratio of 65:35, and dried at 120 °C for 5 min. Each SPC sensor was cut to a dimension of 1 × 0.4″. Further morphological characterization details of the SPC electrodes are available in our previous work^30^.

LIG electrodes were fabricated using an FSL Muse 3D (Full Spectrum Laser) equipped with a 45W CO_2_ laser (λ = 10.6 μm). The lasing process was performed at 9 W of power and a speed of 19.5 cm/s in raster mode at the machine’s default focal distance of 4.65 mm. A layer of Ag paint (Ted Pella Inc.) was applied to the electrode terminals to enhance conductivity, and the RE was coated with Ag/AgCl ink (DuPont™ 5874), as shown in Fig. 1B (left). Morphological characterization of the LIG electrode surface was performed using a Tescan Clara Field Emission Scanning Electron Microscope (FE-SEM). During laser treatment, the polyamide substrate is carbonized, producing a network of porous, few-layered graphene sheets, as illustrated in Fig. 1B (right). This porous architecture greatly enhances the electrode’s surface area relative to flat graphene, thereby increasing the number of available active sites to facilitate electrochemical reactions.

The WE surface for each type of sensor was modified for selective detection of the SARS-CoV-2 spike protein using a multistep electrochemical process (Fig. 1C). For SPC-based sensors, para-aminobenzoic acid (PABA) was electrodeposited via cyclic voltammetry sweeps. The PABA layer was subsequently activated with a mixture of 1-ethyl-3-(3-dimethylaminopropyl) carbodiimide hydrochloride (EDC) and N-Hydroxysuccinimide (NHS) dissolved in 1 mL of 0.5 M 2-(N-morpholino) ethanesulfonic acid sodium salt (MES Na) buffer (pH 6.0) in a 1:1 ratio. The electrodes were then washed with type 1 water and dried under a cold air stream. Following this, the WE was incubated with a 10 μg/mL solution of SARS-CoV-2 spike monoclonal antibody (mAb; reference 4015-D003, Sino Biological) dissolved in 1X PBS (0.01 M phosphate ion, pH 7.4) for 2 h. The electrodes were then rinsed with approximately 5 mL of 1X PBS and dried at room temperature. Finally, the WE was incubated for 1 h at room temperature in a 10 mg/mL solution of BSA dissolved in 0.5 M MES buffer (pH 6.0). In the case of LIG-based sensors, the surface of the WE was modified for selective detection of the SARS-CoV-2 spike protein by direct anchoring of pyrene butyric acid (PBA) at 50 mM, eliminating the need for PABA electrodeposition via cyclic voltammetry. The subsequent steps followed the same protocol as the SPC WE modification process.

Each SPC and LIG WE was characterized using electrochemical impedance spectroscopy (EIS) with a PalmSen4 potentiostat (from Palmsens) and an in-house instrument (SenSARS^30^), using a Ferri/Ferricyanide [Fe(CN)_6_]^-3/-4^ redox probe to measure the ability of the electroactive species to become oxidized and reduced at the modified WE surface. Further details can be found in^30^.

### Viral antigen detection using the immunosensor

Viral load detection was performed by generating an electrochemical impedance spectrum (EIS) through the application of an AC sinusoidal voltage excitation signal (10 mV amplitude) between the WE and the RE across a broad frequency range (10 kHz and 0.02 Hz), at the equilibrium potential of a redox couple (Fe(CN)_6_^4+^/Fe(CN)_6_^3+^). The impedance of the bio-functional layer, representing its resistance to electric current flow under the alternating voltage signal, was used to determine the charge transfer resistance (R_ct_), defined as the difference between the real impedance of the bio-layer and that of the solution. On an unmodified WE, the redox probe easily undergoes oxidation and reduction reactions as its access to the electrode surface is not hindered. While testing with a positive sample, the ions in the redox probe are increasingly obstructed by antigens bound to the mAb sites on the WE, leading to an increase in R_ct_ within the three-electrode system of the SPC. The EIS data between the WE and RE was analyzed using a constant-phase Randles equivalent electrical circuit model^33^ to quantify the physical parameters that describe the WE-fluid interface. For each SPC sensor, EIS was recorded using 50 μl of a 1 mM solution of K_3_Fe(CN)_6_ in PBS 1X (0.01 M of phosphate ion). All SPC sensor measurements were conducted over a frequency range of 20 kHz to 0.02 Hz with an AC signal of 10 mV amplitude. For the LIG electrodes, EIS measurements were conducted over a frequency range of 20 kHz to 1 Hz with an AC signal of 5 mV amplitude between the WE and RE, also at the equilibrium potential of the (Fe(CN)_6_^4+^/Fe(CN)_6_^3+^) redox couple.

### Evaluation of clinical samples

In the FVL study, performance of the electrodes was assessed by placing 0.5 μl of crude Triton-X100 protein extracts from NA (nasopharyngeal Aspirates), OS (oropharyngeal swab), or SA (saliva) samples onto the WE’s surface. For tests conducted at the LSPDV, the samples were applied without Triton-X100 onto the WE’s surface. Clinical samples were incubated on the WE at 5 °C ± 0.5 °C for 5 min, followed by washing with PBS 1X. The electrochemical impedance spectroscopy (EIS) was recorded for each sample to determine the relative concentration of antigenic proteins adsorbed onto the anchored monoclonal antibodies (mAbs). A relative increase of more than 15% in the charge transfer resistance (R_ct_), as determined from the EIS after sample incubation, compared to the reference R_ct_ obtained after electrode functionalization, was considered indicative of a positive infection (see Figure S1 of the Supplemental Material). R_ct_ values < 15% were considered negative. The cutoff value was determined from positive and negative control experiments after incubating 0.5 μl of recombinant SARS-CoV-2 spike proteins (concentration between 1 and 100 fg/mL), Epstein-Barr Virus (EBV) glycoprotein gp350 (Sinobiological ref no. 40373-V08B), and Influenza H1N1 matrix protein 1-M1 (Sinobiological ref no. 40010-V07E) dissolved in PBS 1X at different concentrations.

### Data analysis

Differences in frequencies were evaluated using Fisher’s exact tests and t-test/Wilcoxon rank-sum tests for unpaired data. Concordance was estimated by the Kappa coefficient test and 95% confidence intervals (R software v.3.4.3 and base packages). Interpretation of Kappa values was done as previously described^34^. Likelihood ratios were determined using STATA.

## Results

### Study participants

**Fig. 2:**
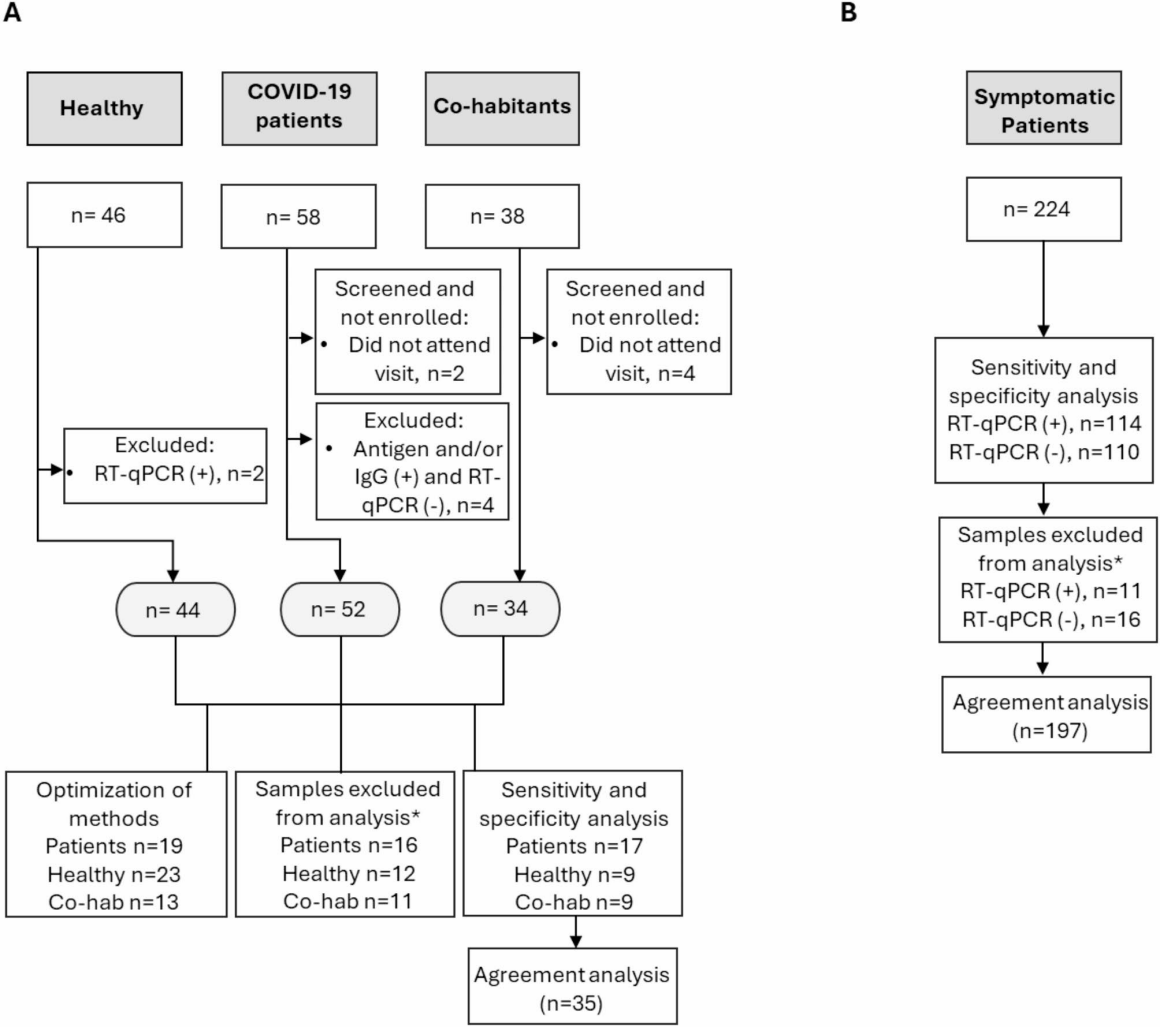
Study participants, eligibility criteria for (**A**) FVL and (**B**) LSPDV. * indicates the samples excluded from the analysis using the immunosensors.

The eligibility criteria for the patients included in both studies are illustrated in Fig. 2A for the FVL case and Fig. 2B for the LSPDV case. A total of 142 adult participants, classified into three study groups, were initially included in the FVL study to evaluate the performance of the SPC-based sensors. However, participants from each study group were excluded for specific reasons: six symptomatic COVID-19 patients were excluded—two due to non-attendance at the sample procurement visit and four due to diagnosis based on antigen or antibody tests but negative RT-qPCR results (see A). Among the 38 co-habitants, four were excluded for not attending the sample collection visit. Additionally, two of the 46 healthy volunteers were excluded due to positive SARS-CoV-2 RT-qPCR results. The samples from the final set of included patients were used for method optimization and validation of the SPC-based sensor validation as detailed in the subsequent sections.

For the study conducted at the LSPDV, all samples analyzed were used to evaluate the performance of the LIG-based sensors. A total of 224 samples from symptomatic patients were measured, comprising 110 samples that tested negative and 114 that tested positive by diagnostic RT-qPCR.

Clinical and sociodemographic data for both case studies are presented in Table S2 (Excel file) of the Supplementary Material.

### Optimization of clinical sample handling and preservation

**Fig. 3:**
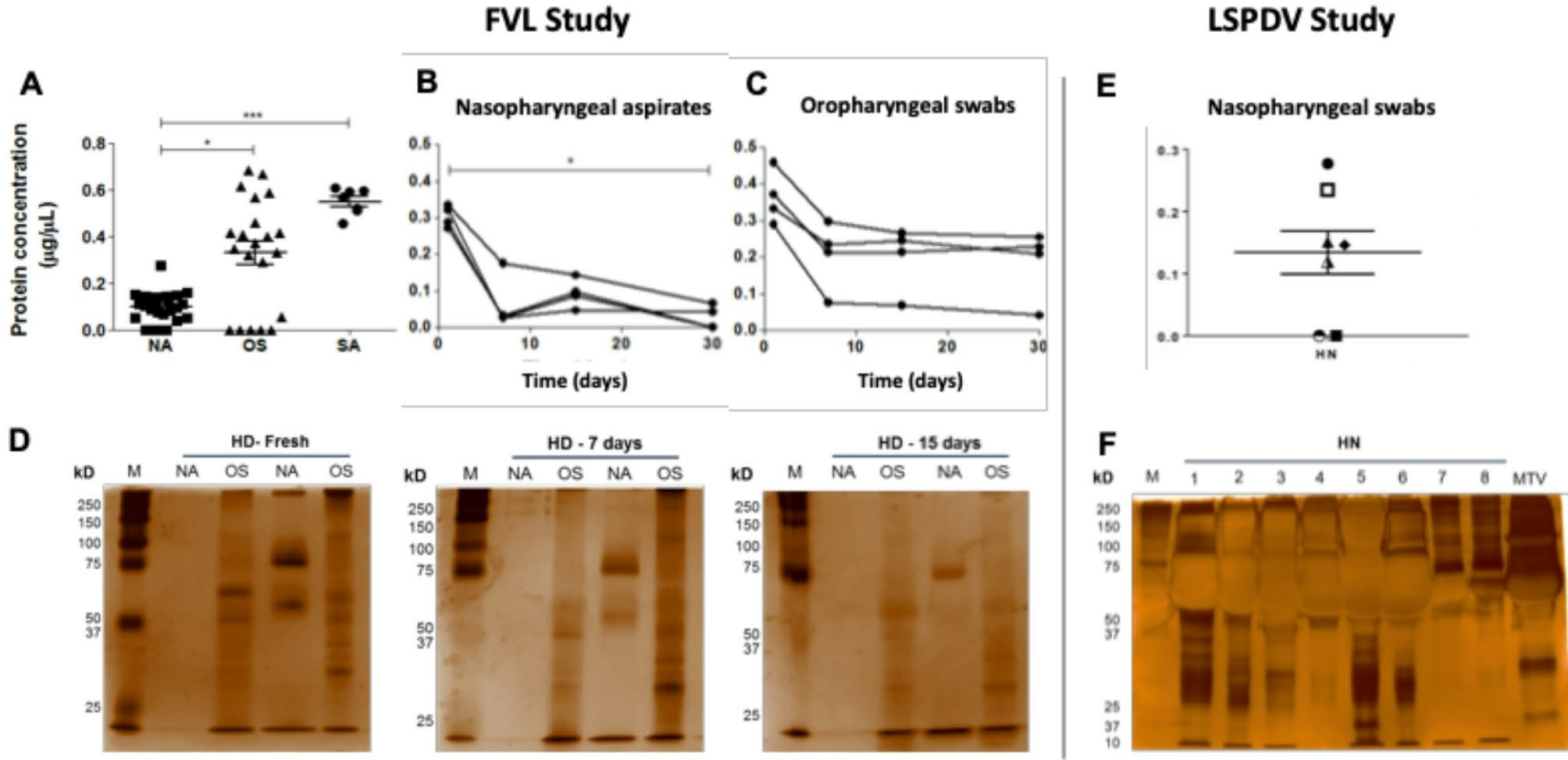
Protein quantification and stability in upper respiratory tract samples for both studies (FVL and LSPDV). (**A**) Determination of proteins by the Bradford method at day one, in 23 healthy participants. OS: oropharyngeal swab (n = 23), NA: nasopharyngeal aspirate (n = 21), SA: Saliva (n = 7). Data are represented as the mean ± SEM and statistical significance was estimated by Kruskal–Wallis test. *: *p* < 0.05, ***: *p* < 0.001. Determination of protein concentration by the Bradford assay. (**B**) Quantification of proteins in nasopharyngeal aspirates samples from four healthy donors (HD) in FVL study. (**C**) Quantification of proteins in oropharyngeal swab samples from two healthy donors in FVL study. Statistical significance was estimated by Kruskal–Wallis test. *: *p* < 0.05. Determination of protein integrity by electrophoresis. (**D**) Polyacrylamide gel electrophoresis on a 12% SDS denaturing gel with silver staining from two healthy donors in FVL study at 1 (fresh), 7 and 15 days. M: marker, NA: nasopharyngeal aspirate, OS: oropharyngeal swab. (**E**) Determination of proteins by the Bradford method from Nasopharyngeal swabs (NS) from 7 SARS-CoV-2 positive participants and 1 healthy participant in LSPDV study, received July 25 of 2022. **F** Polyacrylamide gel electrophoresis on a 12% SDS denaturing gel with silver staining from samples 7 and 8 in LSPDV study. M: marker, NA: nasopharyngeal swab.

A subset of the samples from 55 FVL study participants (19 COVID-19 patients, 13 co-habitants, and 23 healthy donors) were used to optimize study protocols (Fig. 2A), including sample procurement, protein stability analyses, and optimization of sample buffer for readings with the immunosensor. Fresh nasopharyngeal aspirates (NA), oropharyngeal swabs (OS), and saliva (SA) samples from healthy volunteers were used for protein quantification using the Bradford method study. The NA, OS, and SA samples presented an average protein concentration of 0.10 ± 0.06 µg/µL, 0.33 ± 0.23 µg/µL, and 0.55 ± 0.05 µg/µL, respectively (Fig. 3A).

To define the appropriate conditions for sample handling, the maximum time for sample preservation was qualitatively and quantitatively evaluated. Protein integrity and concentration were evaluated in fresh OS and NA samples from healthy donors (n = 4), as well as in aliquots of these samples preserved at -80 °C for 7, 15, and 30 days after procurement. As shown in .

Figure 3B and Fig. 3C, a decrease in protein concentration was observed as early as 7 days of preservation at -80 °C, compared to fresh samples. Despite lower protein concentration measured over time in OS and NA, the protein integrity was preserved, as the band patterns observed for these time-lapse samples were consistent with those detected in the analysis of fresh samples (Fig. 3D). OS show a significantly better protein concentration over time, compared to NA.

We used a subset of 8 samples from LSPDV study participants (7 SARS-CoV-2 positive and 1 healthy donor) to characterize the protein integrity and concentration (evaluated on July 25 2022) for this group. These were samples obtained from NS preserved at 4 °C over a period between February 14 2021 and July 7 2022. As shown in Fig. 3E, the average protein concentration was 0.13 μg/μL ± 0.10. In general, a low average concentration of proteins is observed in these stored samples, which agrees with our previous observations on possible degradation of time-associated proteins storage. In addition, there is a very strong background of proteins from the viral transport medium as seen in Fig. 3F, and this can mask some low abundance antigens in these stored samples.

### Sensitivity and specificity for the study at FVL

The evaluation of the SPC-based immunosensor prototype was performed using 142 samples (NA, SA, and OS) from 74 participants (33 patients, 20 co-habitants of COVID-19 cases, and 21 healthy donors). Of the 142 samples analyzed, 78 (corresponding to samples from 39 participants) were excluded from further analysis due to SPC WE contamination, handling, or degradation issues. One specific problem accounted for most excluded samples: the cross-contamination of the RE by Cl^-^ ions during the incubation of a patient’s sample on the WE led to a shift in the pseudo-reference potential. This was exacerbated by the non-ionic surface in the buffer solution and the interdigitated configuration of the SPC electrodes. Temperature, light, electrolyte concentration, and contamination can all affect the potential of an RE^35^, and the WEs are also susceptible to organic layer denaturing over time. For most cases, immobilized antibodies remained stable after 5 days on the WE, i.e., with no significant signal loss observed. To mitigate some of these deleterious effects, SPC-based electrodes were preserved under dark humid conditions at 4 °C.

Ultimately, samples from 35 participants (17 patients, 9 co-habitants, and 9 healthy volunteers) were used for performance analyses (Table 1). Sensitivity and specificity were evaluated with 64 samples (including NA, OS, and SA). The total number of samples per sample type varied: 13 NA, 29 OS, and 22 SA. The reference standard for the detection of SARS-CoV-2 infection was RT-qPCR of the RdRp and E genes from NA samples. Therefore, sensitivity and specificity analyses were conducted on a per-patient basis, with an additional analysis comparing sample types.

**Table 1:** Diagnostic performance of proposed biosensor for detection of SARS-CoV-2 by samples and study groups in FVL case.

		Proposed Immunosensor									
	RT-PCR*	NA		OS		SA		At least one positive sample per participant^d^		All samples	
		+	−	+	−	+	−	+	−	+	−
Patients^a^	+	4	1	13	1	6	3	15	2	23	5
	−	0	0	0	0	0	0	0	0	0	0
Co-habitants^b^	+	0	2	2	0	2	0	2	0	4	2
	−	0	2	3	3	3	2	4	3	6	7
Healthy donors^c^	+	0	0	0	0	0	0	0	0	0	0
	−	2	2	2	5	2	4	5	4	6	11
All	+	4	3	15	1	8	3	17	2	27	7
	−	2	4	5	8	5	6	9	7	12	18
PPV % (95% CI)		66.7 (22.3–95.7)		**75 (50.9–91.3)**		61.5 (31.6–86.1)		**65.4 (44.3–82.8)**		**69.2 (52.4–83)**	
NPV % (95% CI)		57.1 (18.4–90.1)		**88.9 (51.8–99.7)**		66.7 (29.9–92.5)		**77.8 (39.9–97.2)**		**72 (50.6–87.9)**	
Likelihood ratio (+)		1.71 (0.47–6.30)		**2.44 (1.21–4.90)**		1.60 (0.76–3.36)		**1.59 (1.01–2.52)**		**1.99 (1.24–3.18)**	
Likelihood ratio (−)		0.64 (0.23–1.79)		**0.10 (0.01–0.71)**		0.50 (0.17–1.51)		**0.24 (0.06–1.00)**		**0.34 (0.17–0.71)**	

‘+’: Indicates a measured sample that tested positive for SARS-CoV-2 infection.

‘−’: Indicates a measured sample that tested negative for SARS-CoV-2 infection.

Samples types: nasopharyngeal aspirate (NA), oropharyngeal swab (OS), and saliva (SA).

*The sample type used for RT-PCR as the standard diagnosis was NA.

^a^Patients: Participants with symptomatic SARS-CoV-2 infection.

^b^Co-habitants: Participants asymptomatic co-habitants of patients.

^c^Healthy donors: Participants with no history nor symptoms of COVID-19 infection.

^d^Calculated based on RT-PCR data from NA and sensor results considering positivity in at least one positive sample from each participant. Not validated against subsequent serological (antibody) testing.

^e^Calculated based on RT-PCR data from NA and sensor results considering each independent sample.

Prevalence = Disease present/Disease absent.

Sensitivity = True positive / (True positive + False negative).

Specificity = True negative / (True negative + False positive).

PPV = (sensitivity × prevalence) / [(sensitivity x prevalence) + ((1–specificity) × (1 − prevalence))].

NPV = (specificity × (1–prevalence)) / [(specificity × (1–prevalence)) + ((1 − sensitivity) x prevalence)].

Likelihood ratio (+) = True positive rate / False positive rate = Sensitivity / (1 − Specificity).

Likelihood ratio (−) = False negative rate / True negative rate = (1 − Sensitivity) / Specificity.

In bold. *p* ≤ 0.05 (Fischer’s exact test).

**Fig. 4:**
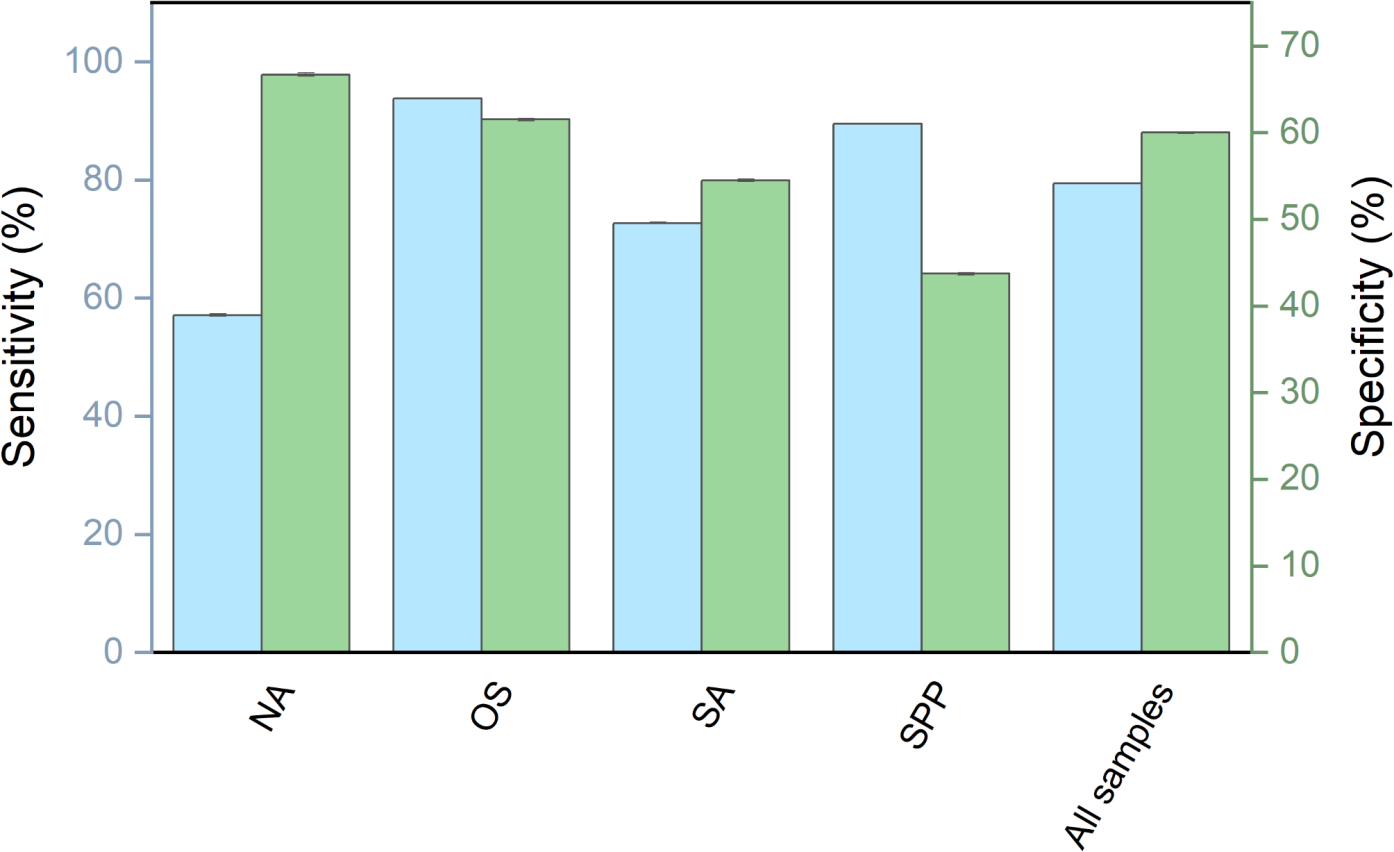
Sensitivity and specificity of proposed biosensor for detection of SARS-CoV-2 by sample type in FVL case. Sample types: nasopharyngeal aspirate (NA), oropharyngeal swab (OS), saliva (SA) and “At least one Sample per Participant” (SPP). Cyan bars indicate sensitivity and green bars specificity.

The average sensitivity of our biosensor for detecting SARS-CoV-2 infection across all sample types (i.e., OS, SA, and NA) was 79.4%, with an average specificity of 60% (Fig. 4). The low specificity was primarily due to discrepancies between RT-qPCR negative and biosensor positive samples (Table 1). A per-sample analysis revealed that the most informative sample for antigen detection using the prototype system was the oropharyngeal swab (OS), which had a sensitivity of 93.8% and a specificity of 61.5% (Fig. 4). This latter value was influenced by RT-qPCR negative and biosensor positive results. Specifically, the biosensor’s specificity was impacted by positive results that were not consistent with the RT-qPCR results in nice participants: four co-habitants of COVID-19 patients and five healthy donors. The clinical records of these participants were reviewed to check for any signs or symptoms of COVID-19 after their participation in the study. At the time of inquiry, five months after the study, none of these participants reported any signs or symptoms of COVID-19. However, no antibody testing was performed on these individuals to confirm asymptomatic infections that might have led to false negatives in RT-qPCR results.

### Sensitivity and specificity for the LSPDV study

A total of 224 samples were analyzed with LIG based electrochemical sensors using a commercial Palmsens4 potensiostat (45 positive and 45 negative samples) and with the SenSARS devices (69 positive and 65 negative samples) developed by our group. Of the 224 samples analyzed, 27 (corresponding to 27 participants) were excluded from further analysis due to LIG contamination, handling, or degradation issues. To decrease cross-contamination of the reference electrode (RE) by Cl^-^ ions during patient sample incubation on the working electrode (WE), the surfactant Triton X-100 was removed. The parameters corresponding to the diagnostic performance are indicated in Table 2.

**Table 2:** Diagnostic performance of the proposed biosensor for SARS-CoV-2 detection in samples from LSPDV case.

	Instrument						
	RT-qPCR	SenSARs		Commercial		Combined	
		+	−	+	−	+	−
Patients	** + **	40	18	31	14	**71**	**32**
	−	10	39	3	42	**13**	**81**
Prevalence (%) (95% CI)		54.2 (44.8–63.6)		50 (39.88–60.12)		**52.3 (45.3—59.3)**	
PPV (%) (95% CI)		80 (68.9–91.1)		91.18 (77.04–96.95)		**84.4 (76.8–92.3)**	
NPV (%) (95% CI)		68.42 (56.3–80.5)		75.00 (62.31–84.48)		**71.1 (63.4–80.0)**	
Likelihood ratio (+)		3.45 (3.23–3.67)		10.33 (8.37–12.76)		**4.98 (4.28–5.81)**	
Likelihood ratio (−)		0.39 (0.18–0.60)		0.33 (0.22–0.50)		**0.36 (0.27–0.48)**	

Significant values are in [bold].

**Fig. 5:**
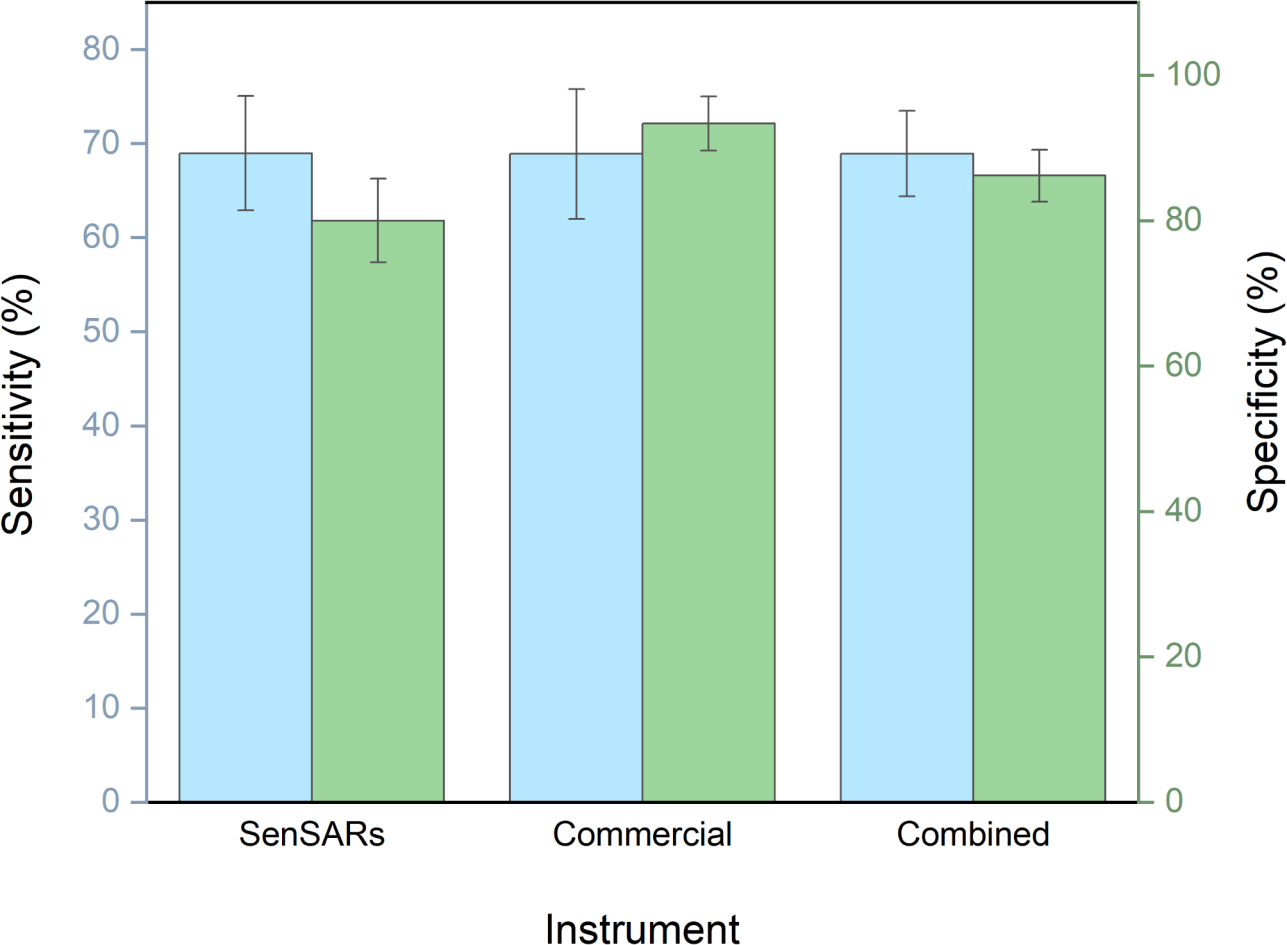
Sensitivity and specificity of the proposed biosensor for SARS-CoV-2 detection in samples from LSPDV case, using two different potentiostats/galvanostats, our in-house designed SenSARS and a commercial unit from Palmsens. The averaged results from both instruments are shown over the “combined” label. Cyan bars indicate sensitivity and green bars specificity.

As shown in Fig. 5 the analysis of the samples in the LSPDV case study revealed that the average sensitivity of our LIG based sensor for detecting SARS-CoV-2 infection in the evaluated samples was 68.93%, with an average specificity of 86.17%.

## Discussion

Technologies that enable frequent, rapid, and accurate screening of individuals at primary care facilities, point of care (POC) sites, and at home are crucial for pandemic containment, especially in resource-limited conditions^36^. Here, we present a proof-of-principle study on the clinical utility of an SPC-based immunosensor for SARS-CoV-2 detection in individuals showing COVID-19 signs and symptoms, who consulted a high-complexity hospital in Cali, Colombia, between October 8th, 2020, and January 1st, 2021. Additionally, a second study validated LIG-based immunosensors that were meant to improve upon the sensitivity of the SPC-based sensors, using samples provided by the LSPDV, collected between February 14th, 2021, and July 25th, 2022.

We demonstrated during the first study (FVL) that among SA, NA, and OS sampling methods, our data shows the highest sensitivity (93.8%) for detecting viral antigens using non-invasive oropharyngeal swabs (OS). This high sensitivity supports its potential implementation across both high and low-complexity clinical and epidemiological settings, offering a viable solution in rural areas where laboratory diagnostics are limited by access to proper health care and infrastructure, which is a common scenario for most of the population in low-middle-income countries.

The biosensor’s specificity, however, could not be fully validated in the first study when compared to the RT-qPCR results. It was later reported by using the same primers and probes used in the first study that the RT-qPCR had a detection limit similar to that of the biosensor (~ 20,000 viral copies/mL)^37^. This similarity between the two methods limited the ability to confirm the biosensor’s accuracy comprehensively. The specificity in the first study was primarily influenced by biosensor-positive results from RT-qPCR-negative samples among clinically healthy volunteers and co-habitants of symptomatic patients. However, a negative RT-qPCR test does not preclude having a subclinical infection, particularly in the absence of further clinical, virological, or serological testing of asymptomatic patients, which was beyond the scope of this study.

The first study highlighted the need for a follow-up study involving more samples and an electrode material that could help improve sensitivity and specificity. We chose synthesizing laser-induced graphene (LIG) electrodes. These electrodes offer significant advantages due to their increased surface area, which, combined with surface modifications, should enhance sensitivity and enable the amplification of analyte signals^38^. Based on findings from earlier research conducted in our laboratory, we optimized laser parameters to adapt LIG for applications like this^39^. As a result, transitioning to LIG electrodes in the second study significantly improved the biosensor’s specificity for SARS-CoV- 2 detection, albeit the sensitivity dropped. We believe this happened due to the choice of ligands used, PBA, which bind non-covalently via pi-stacking to the graphene crystals in LIG. This leads to lower binding energy and higher mobility of the ligands, which can compromise the effective binding of mAb and hence reduce the overall number of antigen binding sites.

The proposed technology demonstrates potential for scalability and cost-effectiveness, as SPC or LIG electrodes can be mass-produced using thick-film screen-printing techniques on flexible PET films or laser engraving polyimide (or other substrates containing monomers with six-carbon membered rings) using a 10.6 or 9.3 μm wavelength commercial CO_2_ laser system, and functionalized with automated liquid handling equipment. A comparison of our technology with other SARS-CoV-2 biosensing approaches (Table 3) shows advancements in the limit of detection, enabling reliable detection even in early stages of infection. While^40^ also reports improved detection times, our work achieves a comparable detection speed with enhancements in LoD and sensitivity. Additionally, the fabrication cost of our electrodes is under $2 USD per unit (see Table S2 of the SI), offering an affordable option that could facilitate cost-effective strategies for monitoring the virus’s progression in human samples, particularly in resource-limited settings. However, further work is required to prevent electrode contamination and degradation, as evidenced by the number of electrodes excluded from analysis – in spite of a relatively high production yield (based on electrical conductivity and visual inspection, see Figure S6 of the SI). Additionally, improving reproducibility through automated production is also a critical point to consider to enhance the reliability of our biosensors.

**Table 3:** Key-features comparison between the proposed sensors against other representative SARS-CoV-2 biosensing technologies.

COVID-19 sensing	Our Work SPC electrode	Our Work LIG electrode	RT-PCR	^41^	^42^	^43^	^40^
Detection Time	5–12 min	5–12 min	2–3 h	< 2 h	> 15 min	30 min	2–3 min
Invasive measurement	NO	NO	NO	NO	NO	NO	NO
Sensing method	Electrochemical (EIS)	Electrochemical (EIS)	Reverse transcription, DNA synthesis and amplification	Electrochemical (DPV)	Electrochemical (DPV)	Electrochemical (DPV)	Electrochemical
LOD	1 fg/mL (77 virions)	1 fg/mL (77 virions)	100 virions	1 copy/μL	15 fg/mL	8 ng/mL	4.12 fg/mL
Sensitivity	93.8%	68.93%	91.4%-94.0%	99%	NA	NA	91.18%
Specificity	61.5%	86.17%	98–99%	> 90%	> 90%	> 80%	> 90%
Selectivity	YES (EPV, H1N1)	YES (EPV, H1N1)	NA	YES (Influenza A, Influenza B)	YES ((S1, BSA, E2 HCV, and CD48)	YES (H1N1)	YES (SARS-CoV-1 S1 or MERS-CoV S1 antigen)
Selective to	Antigen	Antigen	RNA	RNA	Antigen	Antigen	Antigen
Sensing technology	Screen printed carbon (SPC)	Laser-induced graphene (LIG)	NA	RCA amplicons	MIPs	Screen printed carbon black	CNT-FET
Cost per test	< $2 USD	< $2 USD	$100 USD	> $10 USD	NA	NA	NA
Type of samples	AS, HO or SA	AS, HO or SA	AS, HO or SA	AS, HO or SA	AS, HO or SA	SA	SA
Clinically validated	YES	YES	YES	YES	YES	YES	NO

AS: nasopharyngeal aspirate, HO: nasopharyngeal swab, SA: saliva, EIS: electrochemical impedance spectroscopy, RCA: rolling circle amplification, MIPs: molecularly imprinted polymers, HCV: hepatitis c virus, CNT-FET: carbon nanotube field-effect transistor, NA: not available.

## Conclusions

This study demonstrates the design, development, and clinical testing of two low-cost, rapid antigen-based immunosensors for SARS-CoV-2 detection, providing diagnostic results and viral load quantification for under $2. The sensors offer proof-of-principle for scalable, point-of-care diagnostic tools with a time-to-result of under 12 min. The screen-printed carbon-based (SPC) sensor achieved high sensitivity (93.8%) with oropharyngeal swabs (OS) but was limited by reduced specificity (61.5%), potentially influenced by high false positives due to the RT-qPCR reference kit’s limited sensitivity and co-habitant dynamics. Conversely, the laser-induced graphene (LIG)-based sensor with nasopharyngeal swabs (NS) displayed improved specificity (86.17%) but lower sensitivity (68.93%), likely due to a reduction in the total number of effective antigen-binding mAb sites caused by the use of PBA as linker to the laser-induced graphene crystals and steric hindrance within the porous LIG matrix. Both sensor types demonstrated excellent analytical sensitivity and no cross-reactivity with other structural proteins. We demonstrated a 30-day shelf-life (longevity) for bare LIG-based sensors in a separate study^44^. For this study we confirmed consistent and reproducible measurements up to 3 weeks from fabrication and surface functionalization. All sensors (SPC and LIG-based) were fabricated, electrochemical characterized via EIS, and used within the 3 weeks threshold. For all cases, at the time of usage, a blank EIS was applied and used as reference to characterize patient samples (via ΔR_ct_). Overall, changing the electrode material to LIG improved specificity and adaptability to pathogen variants, but seemed to unexpectedly sacrifice sensitivity for the exposed reasons. While the LIG sensor demonstrated superior electrochemical performance, key challenges in production remain, including a relatively lower yield than SPC-based sensors (92.7%, see Fig. S6 of the SI), higher batch variability in terms of initial charge transfer resistance (Fig. S7 of the SI), and a shorter shelf-life (up to 3 weeks). While discrepancies with clinical RT-qPCR results highlight limitations in reference methods and antibody design, these sensors represent promising advancements toward rapid, accessible SARS-CoV-2 diagnostics, especially in resource-limited settings, where cost and time to screening is critical.

## Electronic supplementary material

Below is the link to the electronic supplementary material.

Supplementary Material 1

Supplementary Material 2

## Data Availability

All clinical and sociodemographic data generated or analyzed during this study are included in this published article (and its Supplementary Information files).

## References

[1] Platto, S., Wang, Y., Zhou, J. & Carafoli, E. History of the COVID-19 pandemic: Origin, explosion, worldwide spreading. *Biochem. Biophys. Res. Commun.* **538**, 14–23 (2021). 33199023 10.1016/j.bbrc.2020.10.087 PMC7834510

[2] Naseer, S. et al. COVID-19 outbreak: Impact on global economy. *Front. Public Health* **10**, 1009393 (2023). 36793360 10.3389/fpubh.2022.1009393 PMC9923118

[3] Ahmad, T., Baig, M. & Hui, J. Coronavirus disease 2019 (COVID-19) pandemic and economic impact. *Pak. J. Med. Sci.* **36**, S73 (2020). 32582318 10.12669/pjms.36.COVID19-S4.2638 PMC7306969

[4] Szcześniak, D., Gładka, A., Misiak, B., Cyran, A. & Rymaszewska, J. The SARS-CoV-2 and mental health: From biological mechanisms to social consequences. *Prog. Neuropsychopharmacol. Biol. Psychiatry* **104**, 110046 (2021). 32730915 10.1016/j.pnpbp.2020.110046 PMC7384993

[5] Roemer, C. et al. SARS-CoV-2 evolution in the Omicron era. *Nat. Microbiol.* **8**, 1952–1959 (2023). 37845314 10.1038/s41564-023-01504-w

[6] Markov, P. V. et al. The evolution of SARS-CoV-2. *Nat. Rev. Microbiol.* **21**, 361–379 (2023). 37020110 10.1038/s41579-023-00878-2

[7] Tao, K. et al. The biological and clinical significance of emerging SARS-CoV-2 variants. *Nat. Rev. Genet.* **22**, 757–773 (2021). 34535792 10.1038/s41576-021-00408-x PMC8447121

[8] Dong, Y. et al. A systematic review of SARS-CoV-2 vaccine candidates. *Signal Transduct. Target. Ther.* **5**, 1–14 (2020). 33051445 10.1038/s41392-020-00352-y PMC7551521

[9] Krammer, F. SARS-CoV-2 vaccines in development. *Nature* **586**, 516–527 (2020). 32967006 10.1038/s41586-020-2798-3

[10] Aftab, S., Iqbal, M. Z., Hussain, S. & Hegazy, H. H. Recent advances in nanomaterials-based FETs for SARS-CoV-2 (COVID-19 Virus) diagnosis. *Adv. Funct. Mater.* **33**, 2301007 (2023).

[11] Karuppaiah, G., Vashist, A., Nair, M., Veerapandian, M. & Manickam, P. Emerging trends in point-of-care biosensing strategies for molecular architectures and antibodies of SARS-CoV-2. *Biosens. Bioelectron. X* **13**, 100324 (2023). 36844889 10.1016/j.biosx.2023.100324 PMC9941073

[12] Patel, S. K. et al. SARS-CoV-2 detecting rapid metasurface-based sensor. *Diam. Relat. Mater.* **132**, 109644 (2023). 36575667 10.1016/j.diamond.2022.109644 PMC9780024

[13] Timilsina, S. S., Durr, N., Jolly, P. & Ingber, D. E. Rapid quantitation of SARS-CoV-2 antibodies in clinical samples with an electrochemical sensor. *Biosens. Bioelectron.* **223**, 115037 (2023). 36584477 10.1016/j.bios.2022.115037 PMC9788850

[14] World Health Organization’s (WHO). *Coronavirus Disease (COVID-19) Situation Reports*. Interim guidance on antigen-detecting rapid diagnostic tests (Ag-RDTs). Available at https://iris.who.int/bitstream/handle/10665/342002/WHO-2019-nCoV-lab-testing-2021.1-eng.pdf?sequence=1&.

[15] Younes, N. et al. Challenges in laboratory diagnosis of the novel coronavirus SARS-CoV-2. *Viruses* **12**, 582 (2020). 32466458 10.3390/v12060582 PMC7354519

[16] Kevadiya, B. D. et al. Diagnostics for SARS-CoV-2 infections. *Nat. Mater.* **20**, 593–605 (2021). 33589798 10.1038/s41563-020-00906-z PMC8264308

[17] Ahmed, S. et al. Current advances in immunoassays for the detection of antibiotics residues: A review. *Food Agric. Immunol.* **31**, 268–290. 10.1080/09540105.2019.1707171 (2020).

[18] Rocha, D. S. et al. Disposable and eco-friendly electrochemical immunosensor for rapid detection of SARS-CoV-2. *Talanta* **268**, 125337 (2024). 10.1016/j.talanta.2023.125337 39491949

[19] de Araujo, W. R., Lukas, H., Torres, M. D. T., Gao, W. & de la Fuente-Nunez, C. Low-cost biosensor technologies for rapid detection of COVID-19 and future pandemics. *ACS Nano* **18**, 1757–1777 (2024). 38189684 10.1021/acsnano.3c01629 PMC11537281

[20] Liu, Y. et al. Rapid assays of SARS-CoV-2 virus and noble biosensors by nanomaterials. *Nano Converg.* **11**, 1–24 (2024). 38190075 10.1186/s40580-023-00408-z PMC10774473

[21] Yu, H. et al. Recent advances in field-effect transistor-based biosensors for label-free detection of SARS-CoV-2. *Small Sci.* **4**, 2300058 (2024). 10.1002/smsc.202300058 PMC11935026 40212346

[22] Seo, G. et al. Rapid detection of COVID-19 causative virus (SARS-CoV-2) in human nasopharyngeal swab specimens using field-effect transistor-based biosensor. *ACS Nano* **14**, 5135–5142 (2020). 32293168 10.1021/acsnano.0c02823

[23] Qiu, G. et al. Dual-functional plasmonic photothermal biosensors for highly accurate severe acute respiratory syndrome coronavirus 2 detection. *ACS Nano* **14**, 5268–5277 (2020). 32281785 10.1021/acsnano.0c02439

[24] Cheong, J. et al. Fast detection of SARS-CoV-2 RNA via the integration of plasmonic thermocycling and fluorescence detection in a portable device. *Nat. Biomed. Eng.* **4** (2020). 10.1038/s41551-020-00654-0 PMC8202505 33273713

[25] Kaushik, A. K. et al. Electrochemical SARS-CoV-2 sensing at point-of-care and artificial intelligence for intelligent COVID-19 management. *ACS Appl. Bio Mater.* **3**, 7306–7325 (2020). 35019473 10.1021/acsabm.0c01004

[26] Yakoh, A. et al. Paper-based electrochemical biosensor for diagnosing COVID-19: Detection of SARS-CoV-2 antibodies and antigen. *Biosens. Bioelectron.* **176**, 112912 (2021). 33358057 10.1016/j.bios.2020.112912 PMC7746088

[27] Behera, S. et al. Biosensors in diagnosing COVID-19 and recent development. *Sens. Int.* **1**, 100054 (2020).

[28] Panahi, A., Hassanzadeh, A. & Moulavi, A. Design of a low cost, double triangle, piezoelectric sensor for respiratory monitoring applications. *Sens. Biosensing Res.* **30**, 100378 (2020).

[29] Białobrzeska, W. et al. Performance of electrochemical immunoassays for clinical diagnostics of SARS-CoV-2 based on selective nucleocapsid N protein detection: Boron-doped diamond, gold and glassy carbon evaluation. *Biosens. Bioelectron.* **209** (2022). 10.1016/j.bios.2022.114222 PMC8989705 35430407

[30] Perdomo, S. et al. SenSARS: A low-cost portable electrochemical system for ultra-sensitive, near real-time, diagnostics of SARS-CoV-2 infections. *IEEE Trans. Instrum. Meas.* **70**, 1–10 (2021). 35582002 10.1109/TIM.2021.3119147 PMC8843068

[31] World Health Organization. Laboratory testing for coronavirus disease (COVID-19) in suspected human cases: Interim guidance, 11 September 2020 (World Health Organization (WHO), 2020).

[32] Ramagli, L. S. & Rodriguez, L. V. Quantitation of microgram amounts of protein in two-dimensional polyacrylamide gel electrophoresis sample buffer. *Electrophoresis* **6**, 559–563 (1985).

[33] Hsu, C.-L., Zhang, T., Lo, Y.-H. & Hall, D. A. A low-noise gain-enhanced readout amplifier for induced molecular electronic signals. In *2015 IEEE Biomedical Circuits and Systems Conference (BioCAS)* 1–4 (IEEE, 2015). 10.1109/BioCAS.2015.7348407.

[34] Landis, J. R. & Koch, G. G. The measurement of observer agreement for categorical data. *Biometrics* **33**, 159 (1977). 843571

[35] Ansuini, F. J. & Dimond, J. R. Factors affecting the accuracy of reference electrodes. *Mater. Perform.* **56** (2017).

[36] Dhillon, R. S., Kelly, J. D., Srikrishna, D. & Garry, R. F. Overlooking the importance of immunoassays. *Lancet Infect. Dis.*10.1016/S1473-3099(16)30338-3 (2016). 27676344 10.1016/S1473-3099(16)30338-3

[37] Freire-Paspuel, B. & Garcia-Bereguiain, M. A. Analytical and clinical evaluation of “AccuPower SARS-CoV-2 multiplex RT-PCR kit (Bioneer, South Korea)” and “Allplex 2019-nCoV assay (Seegene, South Korea)” for SARS-CoV-2 RT-PCR diagnosis: Korean CDC EUA as a quality control proxy for developing countries. *Front. Cell Infect. Microbiol.* **11** (2021). 10.3389/fcimb.2021.630552 PMC8223252 34178716

[38] Liaquat, H., Imran, M., Latif, S., Hussain, N. & Bilal, M. Multifunctional nanomaterials and nanocomposites for sensing and monitoring of environmentally hazardous heavy metal contaminants. *Environ. Res.* **214**, (2022). 10.1016/j.envres.2022.113795 35803339

[39] de la Roche, J., López-Cifuentes, I. & Jaramillo-Botero, A. Influence of lasing parameters on the morphology and electrical resistance of polyimide-based laser-induced graphene (LIG). *Carbon Lett.* **33** (2023).

[40] Zamzami, M. A. et al. Carbon nanotube field-effect transistor (CNT-FET)-based biosensor for rapid detection of SARS-CoV-2 (COVID-19) surface spike protein S1. *Bioelectrochemistry* **143**, 107982 (2022). 34715586 10.1016/j.bioelechem.2021.107982 PMC8518145

[41] Chaibun, T. et al. Rapid electrochemical detection of coronavirus SARS-CoV-2. *Nat. Commun.* **12**, 1–10 (2021). 33547323 10.1038/s41467-021-21121-7 PMC7864991

[42] Raziq, A. et al. Development of a portable MIP-based electrochemical sensor for detection of SARS-CoV-2 antigen. *Biosens. Bioelectron.* **178**, 113029 (2021). 33515985 10.1016/j.bios.2021.113029 PMC7826012

[43] Fabiani, L. et al. Magnetic beads combined with carbon black-based screen-printed electrodes for COVID-19: A reliable and miniaturized electrochemical immunosensor for SARS-CoV-2 detection in saliva. *Biosens. Bioelectron.* **171**, 112686 (2021). 33086175 10.1016/j.bios.2020.112686 PMC7833515

[44] Reyes-Loaiza, V. et al. Laser-induced graphene electrochemical sensor for quantitative detection of phytotoxic aluminum ions (Al^3+^) in soils extracts. *Sci. Rep.* **14**, 1–15 (2024). 38459204 10.1038/s41598-024-56212-0 PMC10923804

