# Supplementary material for: Performance assessment of disposable carbon-based immunosensors for the detection of SARS-CoV-2 infections
Source: Sci Rep. 2025 Mar 5;15:7741. doi: 10.1038/s41598-025-92104-7 (PMC11883031; doi:10.1038/s41598-025-92104-7)
Supplement: Supplementary file 1 — Supplementary Material 1 [file 41598_2025_92104_MOESM1_ESM.docx]

**Supplemental Information**

**Performance assessment of disposable carbon-based immunosensors for the detection of SARS-CoV-2 infections**

Olga L. Agudelo^1^, Vanessa Reyes-Loaiza^2^, Lina Giraldo-Parra^3,4^, Mariana Rosales-Chilama^3,4^, Sammy Perdomo^2^, María Adelaida Gómez^3,4^, John W. Rodriguez^2^, Viviana Ortega^2^, Carlos F. Daza Rivera^5^, Diana Galindo^5^, Drochss P. Valencia^2,8^, Mauricio Quimbaya^2^, Simón Plata^2^, Robert Bogdanowicz^6^, Fernando Rosso^1,4^, and Andres Jaramillo-Botero^2,7*^

1. Fundación Valle del Lili, Centro de Investigaciones Clínicas (CIC), Cra 98 No. 18 – 49, Cali 760032, Colombia.
2. iOMICAS, Pontificia Universidad Javeriana, Calle 18 # 118-250, Cali, Colombia.
3. Centro Internacional de Entrenamiento e Investigaciones Médicas (CIDEIM), Cali, Colombia.
4. Universidad ICESI, Cali, Colombia.
5. Secretaria de Salud Departamental-Laboratorio de Salud Pública Departamental del Valle, Cra. 76 #4-70, Cali, Valle del Cauca, Colombia.
6. Faculty of Electronics, Telecommunications and Informatics, Gdansk University of Technology, Narutowicza Str.11/12 | 80-233 Gdansk, Poland.
7. Chemistry and Chemical Engineering, California Institute of Technology, 1200 E California Blvd, Pasadena, CA 91125.
8. Universidad Industrial de Santander, UIS, Carrera 27 Calle 9, Bucaramanga, Colombia

**Corresponding Author:**

**Andres Jaramillo-Botero*

E-mail: ajaramil@caltech.edu

**Screening via the charge transfer resistance of the electrochemical sensors**

Figure S1 depicts the quantification of charge transfer resistance in the proposed electrochemical sensors for screening SARS-COV-2 positive and negative patient from the Electrochemical Impedance Spectrum (EIS).


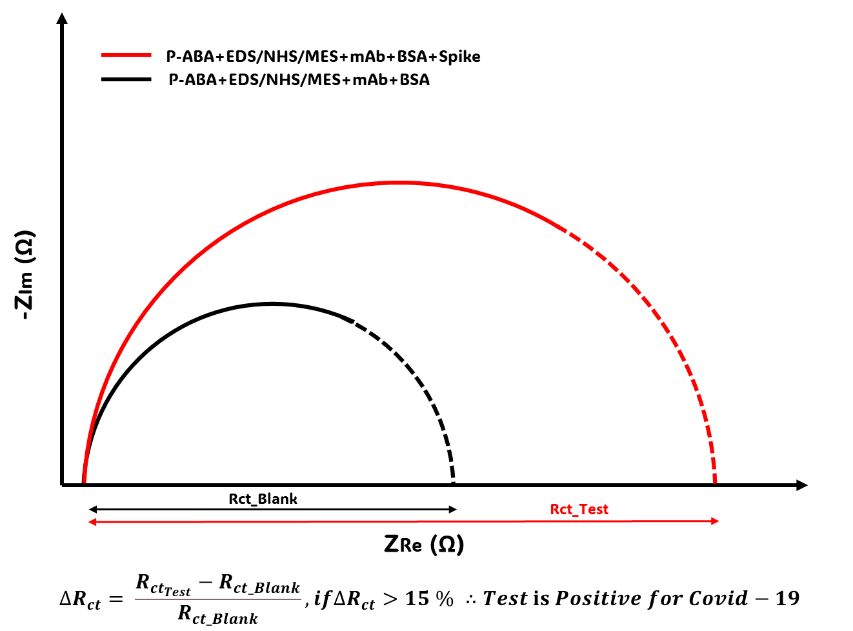


Figure S1. Electrochemical Impedance Spectroscopy (EIS) Nyquist plot showing charge transfer resistance (Rct) for SARS-CoV-2 antigen detection. The plot illustrates the Nyquist response of the working electrode (WE) after sample incubation. The baseline (black curve) represents the Rct of a blank electrode following functionalization with monoclonal antibodies (mAbs) and the BSA layer. The test curve (red) shows an increased Rct value (Rct_Test) after incubation with a positive sample, indicating the presence of SARS-CoV-2 antigenic proteins bound to the anchored mAbs on the WE surface. The relative increase in Rct, calculated as the difference between the Rct of the test and the baseline, was used to determine the positivity threshold. A relative increase above 15% was considered indicative of a positive infection.

**Clinical and sociodemographic data for both studies, FVL and LSPDV**

Table S1. Clinical and sociodemographic characteristics of study participants included in both case studies at Fundación Valle del Lili (FVL) and Laboratorio de Salud Pública Departamental del Valle (LSPDV). Additional data containing the sociodemographics and clinical information specific to the LSPDV study is included in the excel file identified as Table S2 - Supplemental Material - Sociodemographics LSPDV - SARS immunosensors.

|  | **Case study** | | | |
| --- | --- | --- | --- | --- |
|  | **LSPDV** | **FVL** | | |
|  | **Symptomatic patients** | | **Co-habitants** | **Healthy participants** |
|  | **n=224** | **n=58** | **n=38** | **n=46** |
| Age (years): mean (SD) | 38.51 (± 16.7) | 56.13 (± 15.6) | 43.0 (± 19.9) | 39.7 (± 17.7) |
| Female sex: n (%) | 128 (57.1) | 22 (41.5) | 65.7 | 67.3 |
| **Duration of symptoms*** | 5.37 (2.07) | 7.31 (4.76) |  |  |
| Median (range) | 5 (3-10) | 7 (3-10) |  |  |
| 8-13 days: n (%) | 18 (8) | 28 (56) |  |  |
| ≥14 days: n (%) | 4 (1.78) | 18 (36) |  |  |
| >60 days (a subset of the previous row): n (% of total) |  | 4 (8) |  |  |
| **Disease severity:** |  |  |  |  |
| Uncomplicated: n (%) | 199 (88.84) | 10 (17.24) |  |  |
| Mild* | 40 (17.86) | 17 (29.31) |  |  |
| Severe |  | 15 (26.78) |  |  |
| Critical |  | 14 (24.13) |  |  |
| Fatal outcome: n (%) | 1 (0.45) | 7 (7.51) |  |  |
| **Symptoms:** |  |  |  |  |
| Fever: n (%) | 43 (19.19) | 28 (56) |  |  |
| Expectoration: n (%) |  | 6 (12.24) |  |  |
| Cough: n (%) | 68 (30.36) | 29 (59.18) |  |  |
| Difficulty in breathing: n (%) | 6 (2.68) | 20 (40) |  |  |
| Anosmia: n (%) | 30 (13.39) | 8 (16) |  |  |
| Myalgia***: n (%) | 3 (1.33) | 16 (32) |  |  |
| Arthralgia***: n (%) | 1 (0.45) | 8 (16) |  |  |
| Headache***: n (%) | 56 (25) | 10 (20) |  |  |
| Odynophagia***: n (%) | 35 (15.63) | 6 (12.24) |  |  |
| Gastrointestinal***: n (%) | 12 (5.36) | 14(28) |  |  |

**Analytical characterization and calibration of the immunosensors**







Figure S2. Example Cyclic Voltammetry (CV) for (left) SPC-based sensor and (right) LIG based sensor. The results show the LIG-based sensor (right) has superior electrochemical performance compared to the SPC-based sensor (left), based on the higher peak currents, sharper redox peaks (faster electron transfer kinetics), better electron transfer kinetics, and improved sensitivity to the biomolecular interactions between the mAb and the spike antigen. The SPC-based sensor, while functional, has a lower current response and higher surface blocking effects, making it slightly less efficient.







Figure S3. Example Electrochemical Impedance Spectra (EIS) for the (left) SPC-based sensor and (right) LIG-based sensor. The LIG-based sensor has a lower charge transfer resistance, better conductivity, and improved response stability, making it more effective for SARS-CoV-2 detection while maintaining good sensitivity. The SPC-based sensor has a higher baseline impedance and shows a larger increase in charge transfer resistance (R_ct_) upon biomolecular interactions, making it effective for detection but potentially too resistive, which can reduce signal clarity. The LIG-based sensor has lower initial R_ct_ and a more controlled increase in impedance, suggesting better electron transfer kinetics, higher conductivity, and overall improved sensor efficiency.


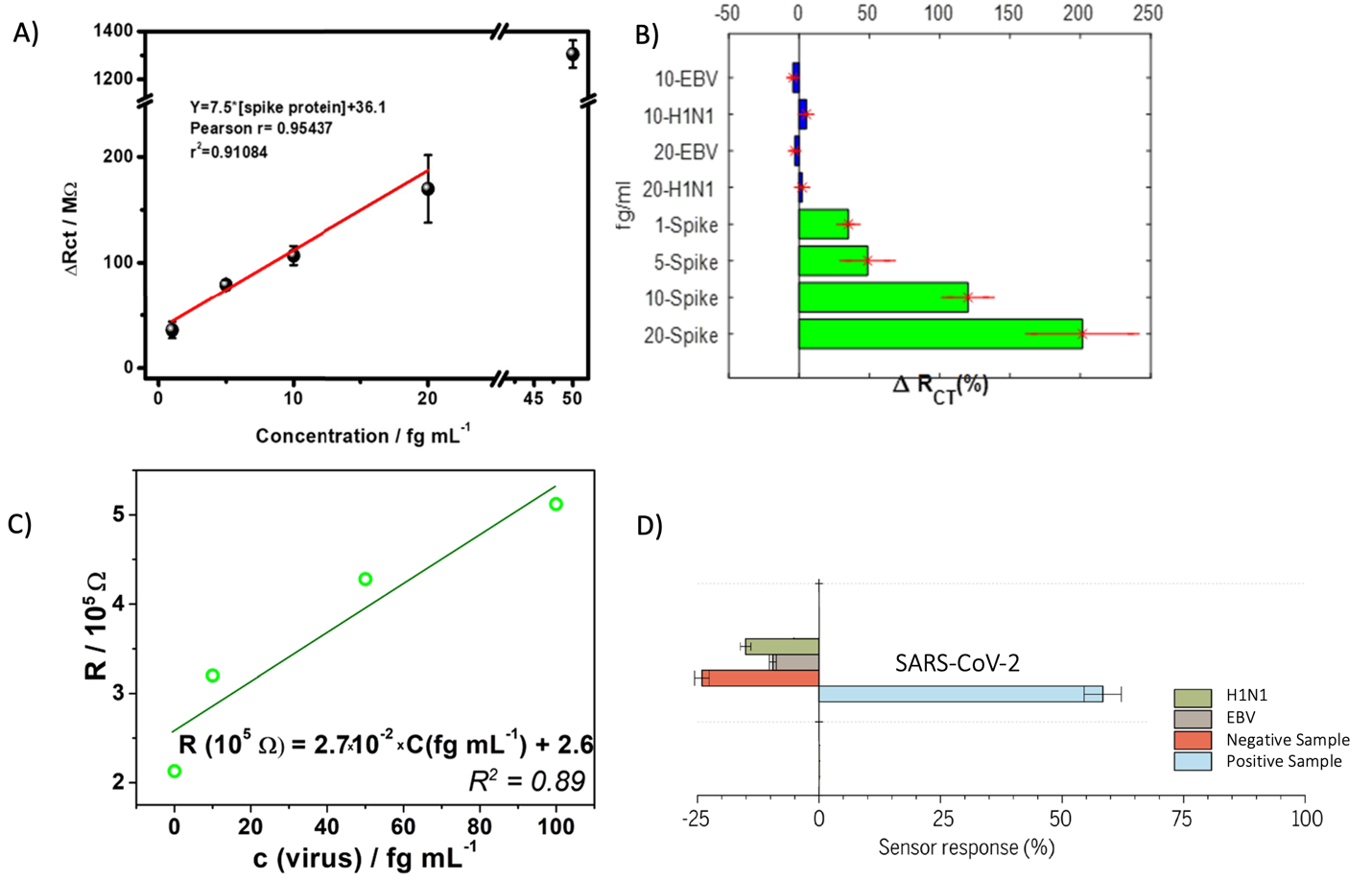


Figure S4. Analytical sensitivity, selectivity, and linearity range of the SPC-based and LIG-based biosensors in this study. A) SPC-based biosensor response as a function of SARS-CoV-2 (S) recombinant protein concentration, B). SPC-based positive and negative control of the SPC-based biosensors (negative controls determine cross-reactivity with other pathogens, EBV and influenza H1N1). Adapted from.^31^ C) LIG-based biosensor response as a function of SARS-CoV-2 (S) recombinant protein concentration, D). LIG-based positive and negative control of the SPC-based biosensors (negative controls determine cross-reactivity with other pathogens, EBV and influenza H1N1). Larger negative response observed for all samples, when compared to the SPC-based sensor.

Figure S4A shows the linear response of our immunosensor to varying concentrations of antigen (recombinant spike protein), ranging from 1 to 20 fg/mL. The signal is given as the absolute change in the charge transfer resistance (ΔR_ct_) across the bio-functional layer of the WE. The change in *R_ct_* calculated for five different blank SPC-based electrodes using a PBS buffer did not exceed an absolute value of 15 %, hence this was chosen as the threshold value for defining negative and positive samples. The mean and standard deviation of the five blank measurements were used, along with the slope of the calibration plot (Figure S4A, analytical sensitivity), to establish the LOD at 1.065 fg/mL, with a 99 % confidence factor. The analytical sensitivity calibration plot shown in Figure S4A was derived from the response signals of three different SPC-based electrodes, tested against samples with varying concentrations of recombinant spike protein. These samples were prepared using a healthy donor saliva matrix and recombinant spike protein in PBS buffer.

Figure S4B shows a high analytical selectivity of our immunosensor to SARS-CoV-2 spike protein, versus other antigenic molecules of pathogens that lead to similar symptomatology, namely, influenza (H1N1) and Epstein-Barr viruses. The EIS was recorded on electrodes incubated with influenza virus (M1 protein of H1N1) and with Epstein-Barr virus (EBV gp350 protein), at 10 and 20 fg/mL dissolved in PBS 1X, 0.01 M at pH 7.4. Results show no significant increase in the *R_ct_* (blue bars in Figure S4B), which confirms no cross-reaction with antigens from influenza and EBV.

Figures S4C and D present the linear calibration fit (based on the EIS R_ct_ results per concentration) and the cross-reactivity analysis with EPV and Influenza H1N1 for the LIG-based sensors.

**Optimization protocol performed for both type of sensors (LIG/SPC)**


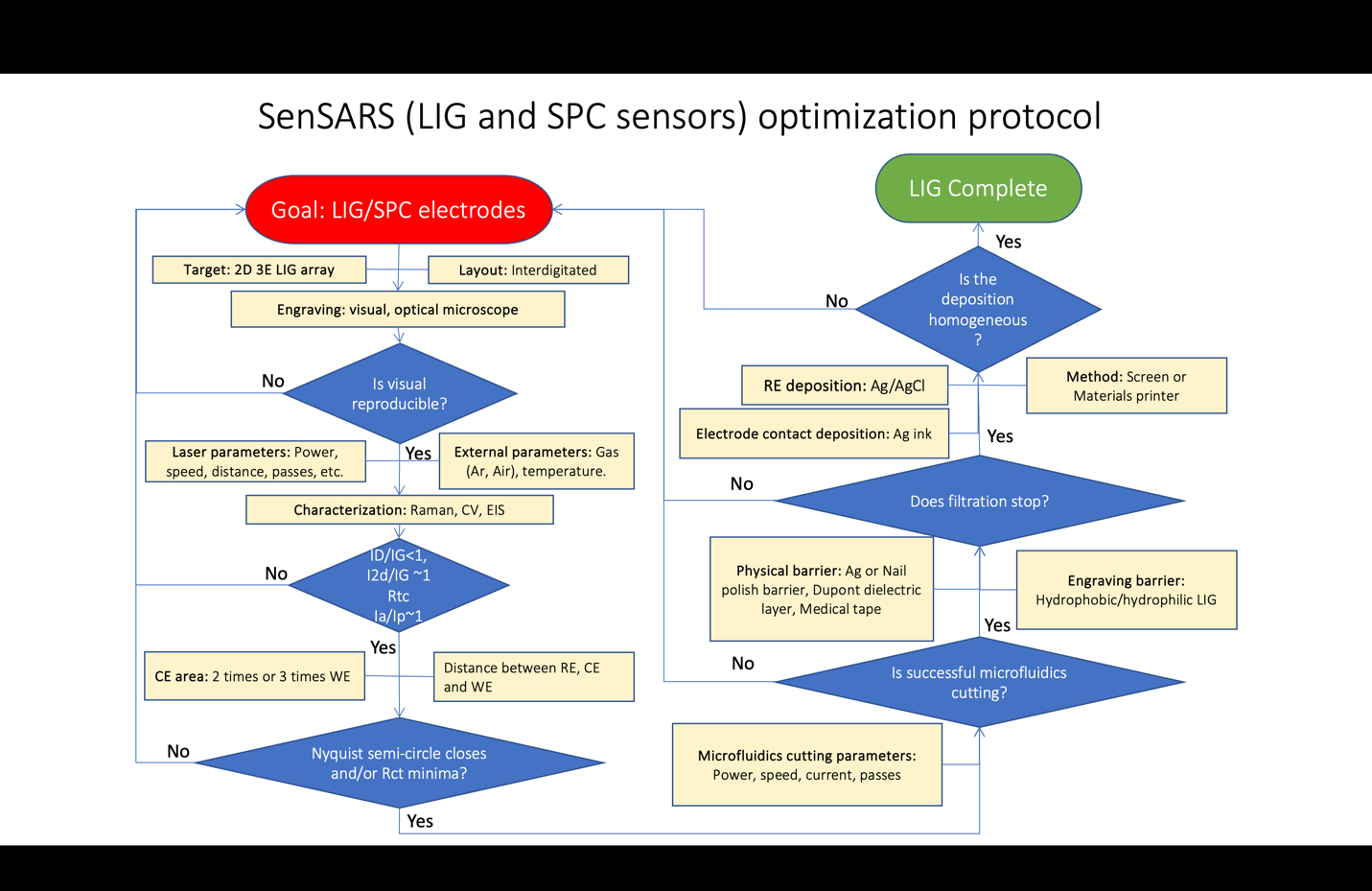


Figure S5. Optimization protocol for screen-printed carbon electrodes and laser-induced graphene electrodes used in SARS-CoV-2 antigen detection. The figure outlines the steps taken to prepare and characterize both electrode types.

**Repeatability and reproducibility testing (LIG-based sensors)**

**
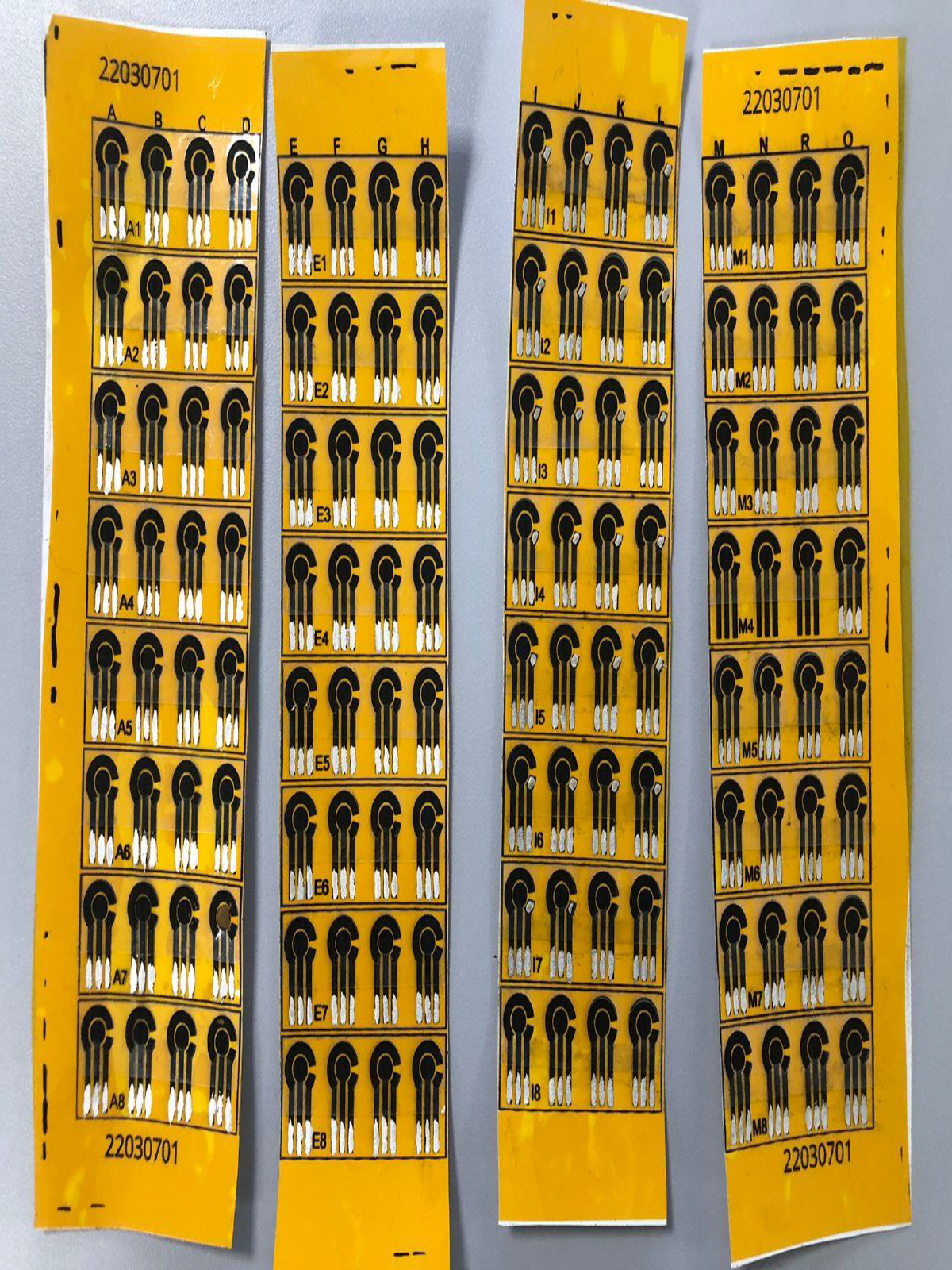
**

Figure S6. Achieved a 92.7% yield in LIG-based sensor production, based on visual inspection and 4-point sheet resistance measurements per electrode. Single PI sheet batch shown above with 8 rows (identified 1-8) and 16 rows (identified A-P), with individual sensors identified by column-row pairs (e.g. A1 for column A row a).


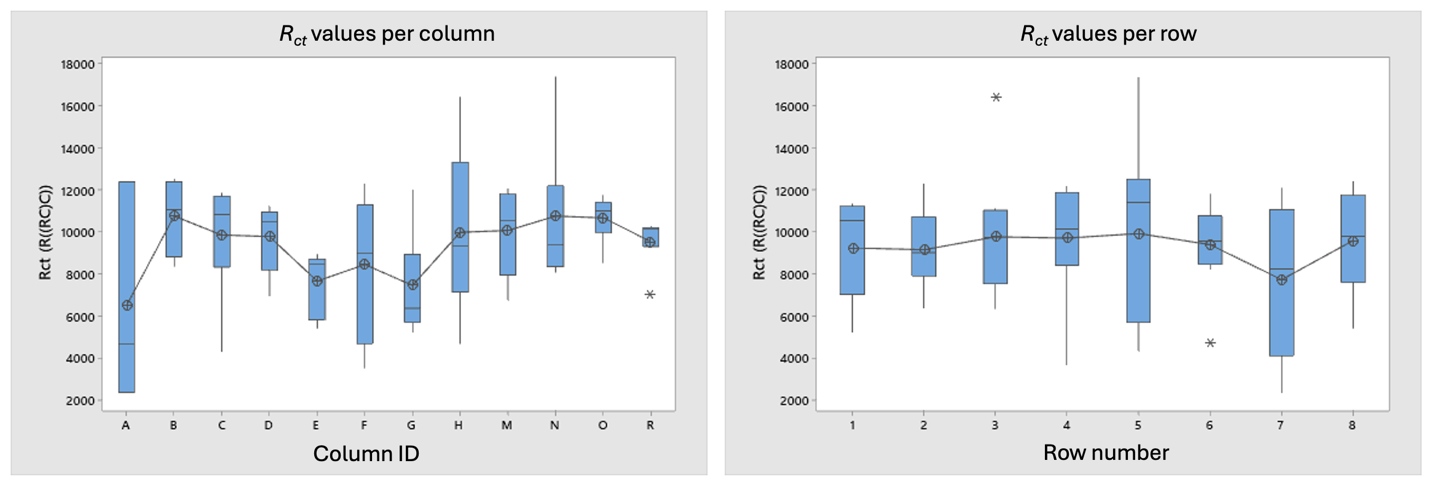


Figure S7. Box plot present the charge transfer resistance (R_ct_) distribution across 128 laser-induced graphene (LIG)-based electrochemical sensors, analyzed by rows (8 rows × 16 sensors). Row-based variation indicates that the process is consistent across rows. Sensors within and R_ct_ of one standard deviation higher than the average R_ct_ per sheet were discarded. Repeatability is moderate and overall fabrication is fairly reproducible.

**Fabrication Costs**

Table S2. Estimated fabrication cost per sensor (SPC-based and LIG-based electrodes).

| **ESTIMATED COSTS PER LIG-BASED SARS-CoV-2 SENSOR, W/OUT LABOR AND EQUIPMENT COST (All values in COP, except total in USD)** | | | | | |  |  |  |
| --- | --- | --- | --- | --- | --- | --- | --- | --- |
| **Reagents** | **Concentration** | **Quantity** | **Units** | **Number of moles, tablets or micrograms** | **Unit cost** | **Price+VAT** | **Total $ COP** |  |
| Methanol | 99.8 | 200.0 | μl |  | $ 145.41 | $ 173.04 | $ 34.61 |  |
| PABA* | 0.02 | 28.5 | μl | 5.70E-07 | $ 830.21 | $ 987.95 | $ 0.08 |  |
| Sodium nitrate | 0.026 | 21.5 | μl | 5.59E-07 | $ 12,470.08 | $ 14,839.39 | $ 0.57 |  |
| HCl | 0.1 | 50.0 | μl | 5.00E-06 | $ 149.76 | $ 178.21 | $ 0.03 |  |
| EDC.HCl** | 0.1 | 2.0 | μl | 2.00E-07 | $ 34,355.34 | $ 40,882.86 | $ 1.57 |  |
| NHS*** | 0.1 | 2.0 | μl | 2.00E-07 | $ 4,393.66 | $ 5,228.46 | $ 0.12 |  |
| MES (pH 6.0) | 0.5 | 204.0 | μl | 1.02E-04 | $ 4,477.40 | $ 5,328.11 | $ 118.05 |  |
| Potassium ferricyanide | 0.001 | 150.0 | μl | 1.50E-07 | $ 1,146.56 | $ 1,364.40 | $ 0.07 |  |
| PBS 1X (0.01M) | 1 | 15.2 | ml | 1.52E-02 | $ 5,272.74 | $ 6,274.56 | $ 95.37 |  |
| Monoclonal antibodies | 10 | 4.0 | μl | 4.00E-02 | $ 87.87 | $ 104.57 | $ 418.28 |  |
| Waste factor |  |  |  |  |  |  | $ 1.33 |  |
| SUBTOTAL |  |  |  |  |  |  | **$ 891.66** |  |
|  |  |  |  |  |  |  |  |  |
| **Substrate and inks** | **Concentration** | **Quantity** | **Units** | **Area or volume** | **Unit cost** | **Price+VAT** | **Total $ COP** |  |
| PET | 10um (3 layers) | 1.0 | cm2 | 9.6774 | $ 52.00 | $ 61.88 | $ 61.88 |  |
| Graphene or carbon ink | 70 mm2 | 1.0 | ml | 0.01000 | $ 2,223.00 | $ 2,645.37 | $ 3,627.86 |  |
| Ag/AgCl ink | 3.5 mm2 | 1.0 | ml | 0.0001 | $ 250.00 | $ 297.50 | $ 0.30 |  |
| SUBTOTAL |  |  |  |  |  |  | **$ 3,690.04** |  |
| **TOTAL** |  |  |  |  |  |  | **$4,581.70** |  |
| **TOTAL SPC-BASED SENSOR USD** |  |  |  |  |  |  | **$ 1.33** |  |
| **TOTAL LIG-BASED SENSOR USD** | **Subtract USD 0.30 from graphene/carbon ink and add USD 0.1875 from Polyimide film** | | | | |  | **$ 1.2175** |  |
| *p-aminobenzoic or para-aminobenzoic acid | |  |  |  |  |  |  |  |
| ** etilcarbodiimide chlorohidrate |  |  |  |  |  |  |  |  |
| ***N-hidroxisuccinimide |  |  |  |  |  |  |  |  |
| Estimate of the variable costs of the reagents used for the functionalization of the SPE sensor. It does not include variable costs for glassware or fixed costs for equipment and personnel. | | | | |  |  |  |  |

**Data availability**

All clinical and sociodemographic data generated or analyzed during this study are included in this published article (and its Supplementary Information files).
